# A future orientation intervention delivered through a smartphone application and virtual reality: study protocol for a randomized controlled trial

**DOI:** 10.1186/s40359-022-01025-x

**Published:** 2022-12-20

**Authors:** Esther C. A. Mertens, Aniek M. Siezenga, Tiffany Tettero, Jean-Louis van Gelder

**Affiliations:** 1 Institute of Education and Child Studies, Leiden University, Leiden, The Netherlands; 2 Department of Criminology, Max Planck Institute for the Study of Crime, Security and Law, Freiburg, Germany

**Keywords:** Smartphone application, Virtual reality, Future self-identification, Future orientation, Goals, Self-defeating behavior, Short-term mindsets, Intervention, Randomized controlled trial (RCT)

## Abstract

**Background:**

Short-term mindsets are associated with self-defeating behaviors, such as delinquency and alcohol use. In contrast, people who consider the longer-term consequences of their decisions tend to report positive outcomes, like feeling more competent and enhanced goal achievement. We evaluate an intervention, FutureU, that aims to stimulate future-oriented thinking, increase goal achievement, and reduce self-defeating behavior, by strengthening people’s identification with their future self. The intervention will be delivered through a smartphone application (app) or immersive Virtual Reality (VR). We test the effectiveness of FutureU for both delivery methods, examine working mechanisms, and identify potential moderators of intervention effects.

**Methods:**

In this Randomized Controlled Trial, a total of 240 first-year university students (*n* = 80 per condition) will be randomized into one of three conditions: (1) a smartphone condition, (2) a VR condition, and (3) an active control condition. We will assess proximal (i.e., future self-identification) and distal intervention outcomes (e.g., future orientation, self-defeating behaviors, goal achievement), user engagement, and examine usage data and goal content. Assessments will take place at baseline, during the intervention, immediately after the intervention, and at 3- and 6-months follow-up.

**Discussion:**

This study will provide information on the effectiveness of the intervention and allows for comparisons between delivery methods using novel technologies, a smartphone app versus immersive VR. Knowledge gained through this study can be used for further intervention development as well as theory building.

*Trial registration* This trial is registered on Clinicaltrials.gov (NCT05578755) on 13 October 2022.

## Background

Considering the future is an important aspect of psychosocial functioning. People who think ahead tend to make more balanced tradeoffs between the immediate and the long-term consequences of their choices [1, 2], and are more inclined to set goals they want to achieve in the future [3]. This results in positive outcomes such as feeling more competent, enhanced goal achievement, saving more, and having better educational records [e.g., 3–4]. In contrast, people who are more focused on the present are also more likely to act on impulse and prefer immediate rewards over larger, delayed rewards [1, 2]. Short-term mindsets are associated with self-defeating behaviors in various domains [e.g., 5]. In order to bolster positive development and goal attainment, and to diminish self-defeating behaviors, the FutureU intervention aims to stimulate future-oriented thinking by increasing future self-identification. The present study examines (1) the effectiveness of the FutureU intervention, (2) working mechanisms, and (3) moderators of intervention effects.

### Intervention theory

The degree to which people consider the future may be related to the degree to which they identify with who they will be in the future, i.e., their ‘future self’ [e.g., 6–10]. People who fail to identify with their future self may underweight or ignore the consequences of their decisions for this remote self [11], similar to how people tend to underweight or ignore the consequences of their decisions to others if left unimagined [12]. The level of identification with one’s future self is assumed to be determined by the extent to which people are able to vividly imagine (i.e., vividness), feel positively towards (i.e., valence), and feel similar and connected to their future self (i.e., related) (in the literature also referred to as ‘future self-continuity’, e.g., [6]). Strengthening the degree to which people identify with their future self may increase their tendency to make choices that favor the needs and wants of the future self over those of the present self [11]. In support of this assumption, empirical research shows that increasing the vividness of the future self is related to positive outcomes in different domains such as reduced delinquency [9, 10], increased exercise behavior [7], and higher savings [4]. Thus, future self-identification seems to positively affect psychosocial functioning.

Our perspective aligns with both identity-based motivation theory [13], and the self-activation hypothesis [14]. According to identity-based motivation theory, our identity functions as a motivator to work towards goals. That is, when people feel a psychological bond with their future self and have integrated an image of their future into their identity, they are more likely to undertake action and make decisions to become this future self and achieve their goals [13]. The self-activation hypothesis posits that behavior is guided by values incorporated into one’s identity. When values are cognitively activated, people will act in line with these values [14]. Hence, people may act according to the values associated with their image of the future when the cognitive representation of the future self is activated.

Our intervention aims to stimulate future-oriented thinking and future self-identification by: (1) actively encouraging participants to imagine their future and the goals they want to achieve in general, (2) explicitly inviting them to contemplate their future self, and (3) enabling them to interact with virtual renderings of their future, i.e., (digitally) aged, self. Vividness of the future and future self plays a particularly important role in our approach. A detailed and vivid image of the future creates the feeling that the future is likely to occur and is close in time, and render people more inclined to make future-oriented choices [15].

We combine this approach with a form of mental time travel commonly referred to as episodic future thinking, which refers to the ability to imagine or simulate *specific* events that may take place in one’s personal future [16, 17]. This has been applied in previous effective interventions addressing diverse behaviors such as impulsive eating [18], food purchases [19], and smoking [20]. For example, in one intervention participants had to vividly imagine and describe a potential future event, including where they were, what they were doing, who were with them, and how they felt. Subsequently, they recalled this event before undertaking a certain task [19]. The more vividly this potential future event was imagined, the larger the intervention effects appeared to be [18], again highlighting the importance of vividness in imagining the future (self).

### Smartphone application versus virtual reality

Given the important role of vividness in both increasing future self-identification and episodic future thinking, new technologies that allow for the transfer of visual information, such as smartphone applications (apps) and immersive Virtual Reality (VR), seem eminently suited as implementation tools. In contrast to traditional interventions, which rely heavily on people’s imaginative abilities [e.g., 19], apps and VR environments provide visual support. For example, age-progressed renderings of participants representing the future self can support people's ability to imagine it. This can reduce the intervention’s cognitive burden as well as the impact of individual differences related to people’s imaginative abilities on intervention effects [e.g., 21].

Both technologies also have unique features that could bolster intervention effects. Apps have few spatial or temporal restrictions: Participants can use them whenever and wherever they want to, including in the home environment [22]. A major advantage of implementing an intervention through an app, therefore, is the possibility of daily exposure to intervention content. To this end, preprogrammed push notifications can, for instance, remind participants to engage with the intervention, or deliver intervention-relevant messages, which, in turn, can increase participants’ treatment adherence [23]. Furthermore, apps create the opportunity to provide psychoeducation via multimedia tools, in which verbal communication can be combined with graphics, text and video, which results in improved recollection and treatment adherence [24].

Immersive VR, in contrast, allows for ‘virtual embodiment’ of the future self and facilitates perspective taking. Virtual embodiment refers to the substitution of an individual’s physical body by a virtual one, with the objective of generating the cognitive illusion that the virtual body is, at least temporarily, one’s own [25, 26]. This illusion of ownership over a virtual body can be induced when participants see their avatar from a first-person-perspective, for instance by seeing its reflection and synchronized movements reflected in a mirror [27]. Characteristics of the avatar that people embody can affect people’s attitudes, behaviors, and cognitions [27]. For example, Banakou et al. [27] found that participants embodying an avatar representing Einstein scored higher on a cognitive task and showed a decrease in age-based discrimination compared to participants embodying an ordinary adult avatar. Furthermore, VR facilitates perspective taking since participants can experience a situation from multiple perspectives, each time embodying a different avatar [25]. Within the FutureU intervention, this means that participants can literally take the perspective of their future self. Creating temporal and psychological distance from daily situations, in turn, can promote future-oriented choices [28].

### The present study

The current study has two aims. First, we aim to evaluate the FutureU intervention in a broad sense. We will assess the extent to which the intervention is able to stimulate future-oriented thinking, increase goal achievement, and reduce self-defeating behavior. More specifically, we will examine proximal intervention effects on outcomes related to future self-identification (i.e., vividness of, valence towards, and relatedness to the future self), and distal intervention effects on both primary (i.e., future orientation, consideration of future consequences, self-defeating behaviors, goal commitment, and goal achievement) and secondary (i.e., self-efficacy, academic results, and impulsiveness) outcomes. In addition, we will examine associations between usage patterns, user engagement, and intervention outcomes of both delivery methods, the FutureU smartphone application and the VR. We will additionally qualitatively analyze content (e.g., specificity, difficulty, and topic) of the goals that are set by participants during the intervention and examine them within the future self-identification framework. Furthermore, we will study whether intervention effects are mediated by increased future self-identification and explore potential moderators of intervention effects, such as personality traits (since personality traits have been linked to the use of new technologies; e.g., [29]). The second aim regards the exploration of differential intervention effects of the two implementation methods. Given that both technologies have their own unique features and advantages, we examine this aim exploratory.

Participants are first-year university students. Prior research suggests people are particularly susceptible to interventions during transformational life events and shifting contexts [14, 30]. During such events, they are more likely to take a ‘big-picture’ view of their lives, which can trigger behavioral changes [30]. Furthermore, a shift in surroundings influences the contextual information people receive, which can affect choices and decisions [14]. For most students, transitioning from secondary school to university is both a transformational life event and a change of context – moving to a new city, a new institution, living independently, being separated from family and close others – making this a relevant period to implement the FutureU intervention.

## Method

### Design

The current study regards an iteration and extension of a prior pilot RCT [see 31] evaluating the first version of the FutureU app. In the present study, the intervention will be examined through an RCT with three conditions: (1) a smartphone condition in which the intervention is implemented via (an iteration of) the FutureU app, (2) a VR condition in which the intervention is delivered through immersive VR, and (3) an active control condition in which participants set goals but receive no further intervention. Ethical approval was obtained from the independent Ethics Board of the Institute of Education and Child Studies at Leiden University (ECPW2021-320). The trial was registered on Clinicaltrials.gov (NCT05578755) on 13 October 2022.

In all conditions, participants start with an intake session. During this session participants give active informed consent, set personal goals, and complete an online questionnaire. In both intervention conditions, an avatar will be created and aged using a machine learning-based algorithm to generate a digital version representing their 10-year older, and hence ‘future’, self (see ‘Avatar Creation’ below).

All participants complete online questionnaires at baseline (i.e., during intake), at weekly intervals during the intervention (assessing a subset of the outcome variables), immediately after the intervention (i.e., three to four weeks after the intake), and at 3- and 6-months follow-up. For the smartphone condition, assessments occur on the 7th day of each of the three week-long modules (i.e., day 7, day 14, and day 21 of the intervention). For the VR condition, assessments occur at the start of the subsequent VR session about a week later. Additionally, future self-identification and the VR experience items are (also) assessed immediately after each VR session. For the control condition, assessments occur at time points parallel to those of the smartphone condition. Questionnaires not completed in time (i.e., within 4 days for the interim measurements, within 8 days for the post measurement, within 16 days for the 3-months follow-up, and within 32 days for the 6-months follow-up) will be treated as missing data.

**Fig. 1:**
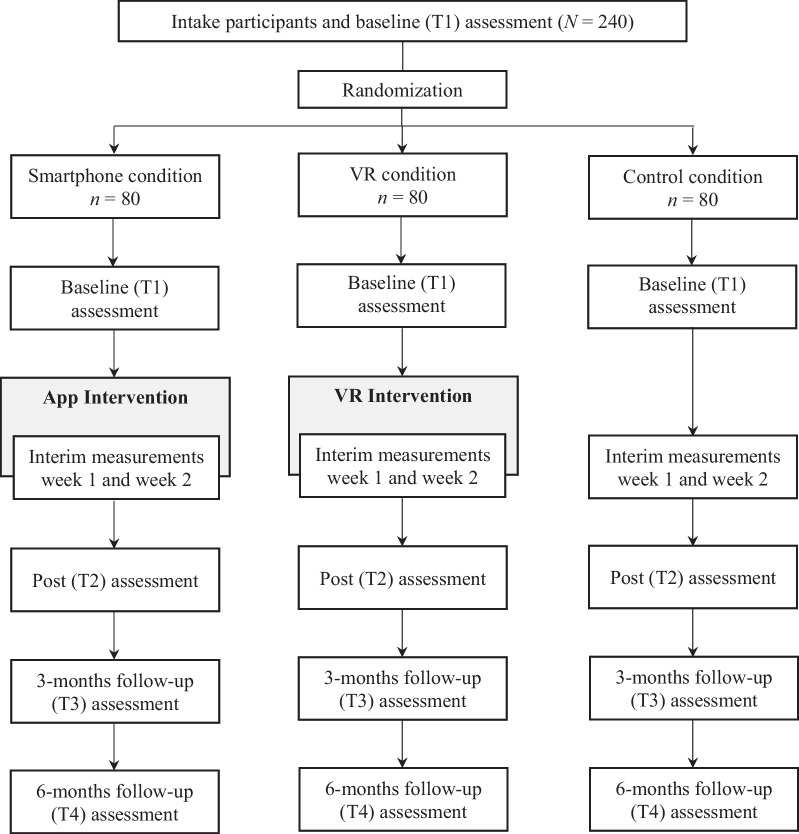
Study flow chart

After receiving participant’ consent, academic results will be requested from the university at the end of the academic year. Participants will receive €35,- or 8 course credits after completing all questionnaires up to the post-measurement. In addition, they will receive €20,- for completing both follow-up questionnaires (see Fig. 1 for the study flow chart).

### Avatar creation

Digital representations of participants, i.e., avatars, are created using various plug-in services and custom software developed specifically for the current purposes.^1^ To create the head of the present self-avatar of participants we use software developed by Avatar SDK, version ‘Head 2.0’ (www.avatarsdk.com). An age-progression service based on machine learning (www.changemyface.com) is then applied to create the future self-avatar.

We will use slightly different pipelines for avatar creation in the smartphone and the VR conditions. In the smartphone condition, the participant takes a ‘selfie’ during the intake using the integrated camera of the participant's smartphone. This image is age-progressed by approximately 10 years via a custom made server and the online service of Change My Face. The aged image is then converted into a 3D digital representation, to create the (avatar of) the future self.

In the VR condition, the (full body) avatar is created at the start of the first VR session in two stages. First, a webcam connected to the computer running the VR is used to make a photo of the participant’s face. For creating the avatar head, the remainder of the procedure is identical to the procedure in the smartphone condition. In the second stage, the body of the VR avatar is created using custom software developed for the research project. Through the use of several sliders, the experimenter adjusts the proportions of a generic male or female virtual body to match the proportions of the participant’s actual body and skin color. The colors of the present- and future self-avatars’ dress are made to differ to emphasize the difference the two.

### Participants and power calculation

We will include first-year students from a university in the Netherlands. Students with epilepsy will be excluded due to increased risk of seizures in VR. To determine sample size an a-priori power analysis was conducted with G*Power. We based our estimate of the effect size on a review of meta-analyses which showed that Cohen’s *d* effect sizes of universal interventions range from 0.23 to 0.58 [32]. Assuming a medium effect size of Cohen’s *d* = 0.40, a significance level of *p* < 0.05, three conditions, and one covariate, a sample size of 199 participants is estimated for a power of 80%. Based on a drop-out rate of 20%, we aim to include 240 participants, i.e., 80 participants per condition.

### Recruitment and randomization

To recruit participants we will distribute flyers in university buildings and use communication channels of the university where the study is conducted, such as the university website and various social media channels. Additionally, we will ask student associations to distribute the advertisement of the study among their members.

Students interested in participating can schedule an appointment for the intake using an online portal. Before the intake, students are assessed for eligibility and randomly assigned to a condition on a 1:1:1 ratio using block randomization with blocks of 9, so that within a block three participants are allocated to each of the three conditions.

### Blinding

Blinding is not possible as all individuals involved will know whether the app or VR will be used or not. However, participants are unaware of the intervention’s content and the study’s hypotheses.

### Conditions

#### Intervention

The intervention starts with participants setting two personal goals during the intake, one goal goal they want to have achieved within a month and one that they want to have achieved in the coming year.^2^ The formulation of these goals is guided by the researcher and follows the SMART-goal model and Zimmerman’s criteria [33]. These guidelines facilitate setting specific, measurable, and challenging but attainable goals – characteristics that are most likely to foster goal attainment [34]. Additionally, each week participants independently set a new goal that functions as the next step towards achieving their month goal (participants in the VR condition receive additional guidance – see ‘VR Condition’ below).

The intervention consists of three modules aimed at (1) instilling a more vivid view of the future self, (2) stimulating future-oriented decision making, and (3) setting and achieving personal goals. Table 1 provides an overview of the three modules, their theoretical foundation, and the translation of the underlying theory into core features and interactions of (the current iteration of) the app and VR.

**Table 1:** Description and Features of the Three Intervention Modules

Module	Theory	Core features Smartphone app	Core features VR sessions
**Future self** Stimulating vividness, familiarity and identification with the future self	• Exposure to and vividness of the future self increases future orientation [46] *Additionally in smartphone intervention:* • Incremental personality theory: The belief that personality can change over time can reduce problematic behaviors [47] • People’s willingness to change on personality traits in socially desirable ways increases after feedback on their current trait levels [48]	• Completion of personal profile of the future self (e.g., work experience, skills, accomplishments) • Current scores on personality traits with an indication of norm scores • Brief animated clip with psychoeducation that personality can change over time • Set desired scores of personality traits of future self • Future self-interaction portal to connect and interact with the future self. The future self asks participants to think about their future self in daily life and provides guided episodic future thinking exercises	• Time travel portal assists mental time traveling in order to ‘pre live’ events • Embodiment of avatar representing future self, bolstering vividness of and identification with the future self • Interview future self about personal profile (e.g., work experience, skills, accomplishments) • A visual grid containing participant’s answers and showing a personal profile of the future self
**Future self perspective** Cultivate future-oriented choices and increase self-insight by distanced perspective taking aiming to stimulate attitudes and behaviors favoring the future self	• People make more future-oriented choices: 1)for others (i.e., Solomon’s paradox [49]) 2) when they have a vivid image of the future self [46] 3) when they can psychologically or temporally distance themselves from the situation (i.e., Construal level theory [28]) • Wise reasoning is enhanced with third-person self-reflection [50]	• Brief animated clip with psychoeducation explaining that people make more future-oriented choices when they can mentally distance themselves from a situation, and when they consider the long-term consequences • Time portal: tool that allows for taking the perspective of the future self to provide advice • Future self-interaction portal: The future self emphasizes that personality can change over time and stimulates decision making with the future self in mind	• Time travel portal and embodiment of future self • Psychoeducation explaining that people make more future-oriented choices when they can mentally distance themselves from a situation, and when they consider the long-term consequences • Ask future self advice on challenges present self experiences. • Switching perspectives is used to mimic a conversation and allows for giving advice • A visual grid containing participant’s answers showing the posed challenges and the future self’s advice
**Goal setting and achievement** Bolster goal setting and achievement by teaching a growth mindset and Mental Contrasting and Implementation Intentions	• Growth mindset: The belief that people’s abilities can develop over time. This mindset aids engagement in thoughts and behaviors to work towards goals [51] • Mental Contrasting and Implementation Intentions [52]: Method in which the desired future is contrasted with the current reality. Obstacles standing in the way of attaining the desired future are identified. Subsequently, a plan is formulated to implement behaviors to overcome the obstacles	• Brief animated clip with psychoeducation that abilities can develop over time • Brief animated clip explaining Mental Contrasting and Implementation Intentions • Practice with Mental Contrasting and Implementation Intentions to work towards goals via filling in a scheme • Writing a letter to the future self with goals • Future self-interaction portal: The future self stimulates taking perspective of the future self (in decision making and in goal achievement) and provides a guided episodic future thinking exercise	• Time travel portal and embodiment of future self • Verbal psychoeducation that abilities can develop over time (i.e., growth mindset) • Practice Mental Contrasting and Implementation Intentions via interviewing the future self, in particular for identification of potential obstacles and formulating plans to overcome these obstacles • A visual grid containing participant’s answers providing a overview of goal, obstacles and plans to overcome the obstacles

#### Smartphone condition

**Fig. 2:**
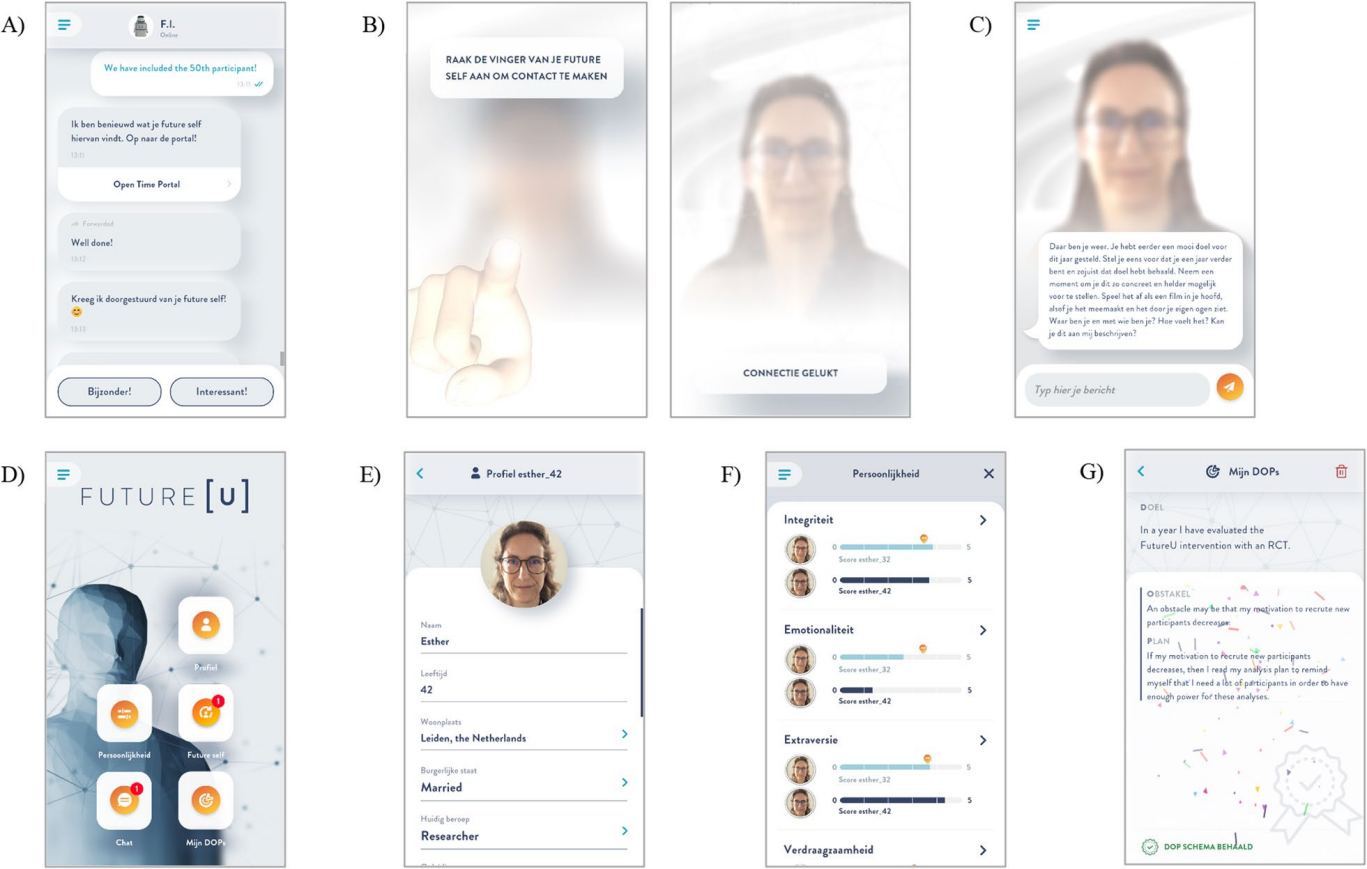
Screenshot of the futureu smartphone application with **A** Chat, **B** the connection mechanic, **C** Future self interaction, **D** Home screen, **E** Personal profile, **F** Personality menu, **G** Goal scheme

Participants receive a daily push notification reminding them to open the app. When participants click on the push notification, they are directed to a chat function integrated in the app where they interact with the app’s chatbot FI. Through scripted messages, FI provides psychoeducation, asks (multiple choice or free text entry) questions designed to trigger thinking about the future, and gives instructions for the interaction or assignment of that day (e.g., set desired personality scores of the future self, take the perspective of the future self). Additionally, FI occasionally engages in small talk or sends images to keep the interaction engaging (see Fig. 2A). On a regular basis (11 interactions in total, i.e., roughly every other day of the intervention period), participants also receive a push notification from their future self. Clicking on this notification leads the user to a ‘future self-interaction’ menu in the app. Participants ‘connect’ with their future self by touching the (virtual) finger of their future self on the screen of their smartphone. Subsequently, the app generates a pulse, the screen unblurs, and the avatar of the future self becomes (more) clearly visible (see Fig. 2B).^3^ The purpose of the interactions with the future self is (1) to help instill a more vivid image of the future self, (2) to encourage participants to think about their future self in their daily life, and (3) to strengthen the sense of connection with the future self (see Fig. 2C). The scripted interactions contain elements of psychoeducation (e.g., incremental personality theory, temporal distancing), requests by the future self (e.g., “When making a decision today, try to make this decision from my [i.e., the future self’s] perspective.”), and guided episodic future thinking exercises (e.g., imagining their graduation day).

Participants use the app on a daily basis for a period of 21 days allowing them to complete all three week-long intervention modules. Over the course of the intervention period the different features of the app (i.e., personal profile of the future self, personality overview, goal attainment schemes; see Figs. 2D–G) are consecutively unlocked. The daily interactions take approximately five minutes or less to complete, favoring frequency of exposure to the intervention content over length of contact.

#### VR condition

**Fig. 3:**
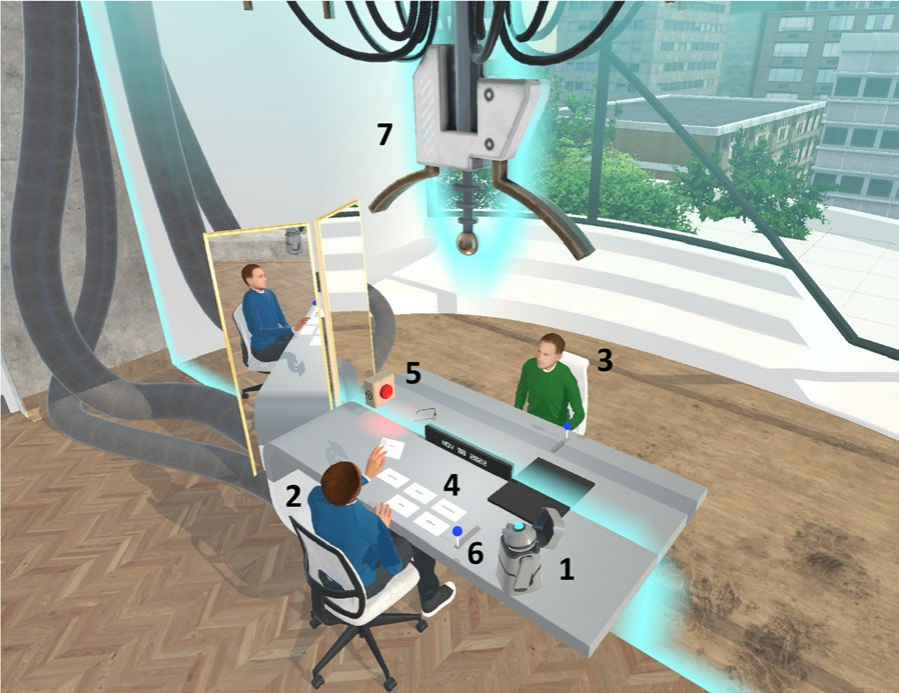
General overview of the futureu VR environment. 1 = Robot controlled by researcher; 2 = Present self-avatar; 3 = Future self-avatar; 4 = Cards with questions; 5 = Recording light; 6 = Handle to travel through time; 7 = Time machine

The VR sessions take place in an immersive virtual reality environment consisting of a large room ostensibly on the top floor of a high-rise building with a table standing in the middle. The avatar representing the future self and the avatar representing the present self sit at opposite sides of the table (see Fig. 3). A time machine which, according to the narrative of the intervention, allows for time travel hangs above the table.

Each VR session, participants alternate between embodying the avatar representing their present self and the avatar representing their future self. The sessions are guided by a virtual robot, FI, which hovers in sight of the participant and is controlled by the researcher. FI provides brief episodes of psychoeducation (e.g., temporal distancing, Mental Contrasting and Implementation Intentions) and explains the controls and interactions to the participant. Interactions are (mainly) card based. When participants embody their present self, they are presented with (multiple) cards with questions laid out on the table. Subsequently, they read the questions out loud one by one. This is recorded by the VR system. Then, by pressing a virtual lever, participants ‘travel through time’ and embody their future self on the other side of the table. The recorded questions are played back and participants respond to the questions they had just read out loud from the perspective of their future self. These responses are also recorded and played back when the participant time travels back to embody the present self-avatar again.

Each session contains one or two rounds of structured interaction, in which participants read out loud the questions on the cards,^4^ and one round in which they are free to ask their own questions to their future self. In line with the app, the scripted questions are designed to (1) create a vivid image of their future self, (2) trigger thinking about the future, (3) strengthen the connection with their future self, and (4) practice with temporal and psychological distancing by switching perspectives. To facilitate reflection on session content as well as self-reflection, participants’ answers are noted in keywords by the researcher and presented to the participant on a virtual grid. At the end of each session, participants shortly reflect on their answers. The grid content is also emailed to the participants after the session with the objective of stimulating reflection outside of the VR environment. Sessions one and two end with FI inquiring about the participants’ goal for the week and what first step they can take towards achieving this goal. Session three ends with asking participants what their first step towards their goal for the year will be. Asking participants about concrete actions they can undertake towards achieving their goals serves as a ‘call to action’, activating participants to actually undertake steps towards achieving their goals.

Before participants go into the VR environment, they set a new goal for the coming week. As during the intake, goal formulation is guided by the researcher to ensure it is in line with the SMART-goal model and Zimmerman’s criteria. Each of the three VR sessions will last about 30 min and will be guided by a trained researcher who follows a standardized script.

#### Control condition

Following the same procedure as in the two intervention conditions, during the intake and guided by the researcher, participants set a goal for the coming year and for the coming month. They will also independently set weekly goals as steps towards achieving their month goal. Setting specific, measurable, and challenging goals has been related to increased positive outcomes [e.g., 34]. Therefore, this active control condition enables us to analyze the effects of the intervention beyond the potential effects of goal-setting.

### Measurements

Table 2 provides an overview of the concepts, instruments, and assessment time points.

**Table 2:** Overview concepts, instruments and assessment time points administered

Concept	Instrument	#Items	*T*1	Int	*T*2	*T*3	*T*4
Future self-identification	Based on Hershfield et al.[35] and Van Gelder et al. [9]	8	x	x	x	x	x
Future orientation	Future Orientation Scale [1]	15	x	x^1^	x	x	x
Consideration of future consequences	Consideration of Future Consequences questionnaire [36, 37]	9	x		x	x	x
Self-defeating behavior	Self-defeating behavior list based on Van Gelder et al. [9]	15	x	x	x	x	x
Goal commitment	Goal Commitment Questionnaire [38]	7	x		x	x	x
Weekly goal achievement	Self-developed	3		x	x		
Monthly goal achievement	Self-developed	3			x		
Self-efficacy	General Self-efficacy Questionnaire [39]	10	x		x	x	x
Academic results^2^	University records	–					x
Impulsiveness	Barratt Impulsivity Scale 15 item version [40]	15	x		x	x	x
Think about future (self)	Self-developed	2	x	x	x	x	x
Personality	HEXACO-60 [41, 42]	60	x				x
App experiences^3,4^	TWEETS [43] and self-developed items	30		x	x		
App usage^4^	Log data app	–		x			
VR experiences^5^	VR related concepts (e.g., embodiment, presence, Proteus effect) Based on previous studies [27, 44, 45] and self-developed items	34		x			
VR usage^5^	Log data of the VR environment	–		x			
Goals	Qualitative coding	–	x	x	x		

*T*1 = baseline, Int. = interim assessments, *T*2 = post measurement, *T*3 = 3-months follow-up, *T*4 = 6-months follow-up, ^1^On the Int. a subset of 6 items is assessed, ^2^University records will be requested at the end of the academic year, ^3^Self-developed items are only assessed at *T*2, ^4^Only assessed in the smartphone condition, ^5^Only assessed in the VR condition

#### Proximal outcomes

The extent to which people identify with their future self (i.e., future self-identification) will be assessed with three scales representing different aspects of this concept: Vividness, valence, and relatedness.

Vividness of the future self, i.e., the degree to which people can imagine their future self vividly will be assessed with five items (e.g., “I have a clear image of myself in 10 years from now”) based on Van Gelder et al. [9] answered on a 7-point Likert scale (1 = *completely disagree* to 7 = *completely agree;* α $\ge$ 0.87).

Valence towards the future self, i.e., the level of positive feelings towards the future self will be measured with one item: “How do you feel when you think about your future?” [35]. The item is answered with the Self-Assessment Manikin ranging from negative to positive feelings on a 9-point scale.

Relatedness to the future self, measuring the extent to which people feel connected and similar to the future self, will be assessed with the 2-item Future Self-Continuity Measure by rating the extent to which two circles, representing the present and future self, overlap on a 7-point scale [35].

#### Distal outcomes

##### Primary outcomes

Future orientation is measured with the Future orientation Scale [1] measuring time perspective, anticipation of future consequences, and planning ahead. The 15 items of the scale consist of a present-oriented and a future-oriented statement (e.g., “Some people spend very little time thinking about how things might be in the future, but other people spend a lot of time thinking about how things might be in the future.”). Choosing the present-oriented statement results in a score of 1 (= *completely true*) or 2 (= *a little bit true*) and choosing the future-oriented statement results in a score of 3 (= *a little bit true*) or 4 (= *completely true*; *α* = 0.80). For the interim assessments, a selection of six items, based on factor loadings and face validity, will be used.

Consideration of future consequences, i.e., the degree to which people take immediate versus distant consequences into account in potential behaviors, will be assessed with the Consideration of Future Consequences questionnaire [36, 37] consisting of 9 items (e.g., “I consider how things might be in the future.”) answered on a 5-point Likert scale (1 = *completely disagree* to 5 = *completely agree*; *α* = 0.81).

Self-defeating behavior, that is, behaviors with immediate gains though long-term costs, will be measured with 15 items representing different self-defeating behaviors (e.g., “How often in the past week have you missed school or work?”) based on the measure of Van Gelder et al. [9]. The items are answered on a 5-point Likert type scale (1 = *never* to 5 = *more than 10 times*).

The degree of commitment to the goal participants set for the year will be measured using the Goal Commitment Questionnaire [38], which consists of seven items (e.g., “I think this goal is a good goal to shoot for.”) answered on a 7-point Likert scale (1 = *completely disagree* to 7 = *completely agree*; *α* = 0.71).

Weekly and monthly goal achievement will be assessed with three items developed for the purposes of the study: “I have often thought about my goal”, “I have work hard towards my goal”, and “I have achieved my goal”. Items are answered on a 5-point Likert scale (1 = *completely disagree* to 5 = *completely agree*).

##### Secondary outcomes

Self-efficacy, i.e., people’s sense of competence to effectively deal with life’s stressors, will be measured with the General Self-efficacy Questionnaire [39] consisting of 10 items (e.g., “I can always manage to solve difficult problems if I try hard enough.”) answered on a 4-point Likert scale (1 = *completely disagree* to 4 = *completely agree*; range *α* = 0.75–0.91).

Academic results, i.e., grade point average, will be requested from the university at the end of the academic year after participants’ consent.

Impulsiveness, indicated by lack of impulse control on planning, motor, and attention, will be assessed with the Barratt Impulsiveness Scale short form [40] consisting of 15 items (e.g., “I do things without thinking.”) answered on a 4-point Likert scale (1 = *completely disagree* to 4 = *completely agree*; *α* = 0.79).

#### Other measurements

The extent to which participants think about their future and their future self will be assessed with 2 items: “How often in the past week have you thought about your future?” and “How often in the past week have you thought about your self in the future?”. The items are answered on a 5-point Likert type scale (1 = *never* to 5 = *more than 10 times*).

Personality will be measured with the Dutch version of the 60-item HEXACO-60 Personality Inventory [41, 42]). The HEXACO-PI measures six personality dimensions, Honesty-Humility, Emotionality, Extraversion, Agreeableness, Conscientiousness, and Openness to Experience, with 10 items each (e.g., Honesty-Humility: “I am an ordinary person who is no better than others.”; Conscientiousness: “When working, I often set ambitious goals for myself.”) answered on a 5-point Likert scale (1 = *completely disagree* to 5 = *completely agree;* range *α* = 0.71–0.79).

App engagement will be assessed using the Twente Engagement with Ehealth Technologies Scale (TWEETS [43]) consisting of 9 items (e.g., “This app is part of my daily routine.”; *α* = 0.86–0.87) answered on a 5-point Likert scale (1 = *completely disagree* to 5 = *completely agree*) and 21 self-developed items measuring engagement with the app’s specific features (e.g., “I recognized myself in the avatar.”) answered on a 7-point Likert scale (1 = *completely disagree* to 7 = *completely agree*).

App usage will be measured through passively collected log data of the app, such as the number of times participants accessed the app, how long and how often they engaged with specific modules, and the daily and total time spent using the app.

VR experiences of participants will be assessed with the following scales: Embodiment (i.e., experiencing the virtual avatar as the own body; 5 items, 4-point Likert scale; based on Banakou et al. [27]), the Proteus effect (i.e., taking over characteristics associated with the embodied avatar; 3 items, 4-point Likert scale; self-developed), presence (i.e., having the feeling of actually being present in the virtual environment; 4 items, 4-point Likert scale; based on Hartmann et al. [44]), engagement (i.e., feeling engaged in the VR task; 14 items, 4-point Likert scale; based on O’Brien et al. [45]). Additionally, after the last VR session we will measure the way participants experienced their avatar (i.e., identification and recognition in the avatar of the future self; 7 items, 7-point Likert scale; self-developed).

VR usage will be measured with passively collected log data of the VR sessions that provide a description of the time spent in each of the two avatars and the total time spent in the VR environment.

The content of the goals set by participants at the start and during the intervention will be qualitative assessed, coding aspects such as goal specificity, goal difficulty, topic of the goal, and goal type.

##### Data management

All members of the research team will sign a confidentiality statement, become familiar with data management and -storage procedures, and will have access to the data. Data will be collected through online questionnaires and stored on the secured servers of Leiden University, which are backed up regularly. Data quality will be monitored by incorporating attention checks in questionnaires.

##### Statistical analyses

The data will be analyzed using an intention-to-treat approach which implies that all randomized participants will be included in the analyses regardless of whether they actually completed the intervention or not. The three conditions will be compared on age and gender to examine potential differences at baseline. In case of baseline differences between the conditions, we will correct for them in the analytical models.

To test the effectiveness of the intervention and investigate differences in intervention effects between the two implementation methods, we will use regression models and ANCOVAs with the baseline measure of the concerned outcome as a covariate. Mediation will be examined with regression analyses. We will analyze whether the intervention conditions are associated with changes in the mediators (e.g., vividness, valence, and relatedness) and whether these changes, in turn, are related to changes in the intervention outcomes. Moderation of intervention effects will be analyzed with interaction effects in regression models and multigroup models.

Furthermore, we will study how (fluctuations in) usage patterns and engagement levels in an app and VR change over time and relate to intervention outcomes in an exploratory fashion using regression analyses possibly complemented with additional analyses (e.g., latent profile models). We will also qualitatively examine participants’ goal content, coding aspects such as the degree of specificity, the difficulty level, and the topic. Additionally, we will explore the role of goal content within the future self-identification framework. More specifically, we will relate aspects of goal content to the future self-identification scales (i.e., vividness, valence, and relatedness) over time, and examine the potential mediating role of self-efficacy in this context. Both will be analyzed with regression analyses.

## Discussion

The present study tests a novel intervention, FutureU, which aims to stimulate future-oriented thinking, increase goal achievement and reduce self-defeating behavior by strengthening people’s future self-identification. We will evaluate FutureU by examining its effectiveness, its working mechanisms, and potential moderators of intervention effects. Additionally, we will explore usage patterns, engagement levels, and goal content within an intervention context. The intervention will be delivered through an app and immersive VR. Both technologies have their own advantages for intervention implementation, though they are rarely simultaneously included in research designs. Our three-arm RCT provides a unique opportunity to compare these two technologies as implementation strategies.

Although the use of technology for intervention implementation is appealing, it also comes with challenges. First, if people consider the app or VR as unappealing, they may stop using it or feel less engaged [23]. In order to make the app and VR environment attractive and engaging we conducted extensive user-tests, using questionnaires as well as qualitative interviews, and a large-scaled pilot RCT examining an earlier version of the FutureU app intervention [31] in which we collected data regarding user experiences, app use, and intervention effects. Earlier versions of the virtual environment have been used in prior research as well [10]. Based on feedback on prior iterations, we iterated both the app and the VR. In the current RCT, we will collect usage data to examine to what extent treatment adherence (e.g., using the app each day, participating in all VR sessions) is a concern. Second, drop-out rates tend to be relatively high in studies using new technologies [23]. In addition, our study includes multiple measurement points which, although strengthening our study design, also increases participant burden. Therefore, besides the substantial effort we have put into the development of an engaging intervention, participants also receive compensation (financially or with course credits) for their participation.

In conclusion, this study will provide an extensive evaluation of FutureU, as well as a comparison of implementation strategies using different types of technology. Comparing implementation via different technologies will provide initial insights into which technological features may bolster intervention effects and whether implementation via a combination of technologies would be interesting to examine further. Even though we test the effectiveness of the intervention among university students, FutureU (in current or iterated form) has been conceived to also be relevant for a broad range of other populations. A stronger identification with one’s future self has already been associated with positive effects on various domains (e.g., savings [4], health [7], and delinquency [9, 10]). Thus, the knowledge gained with the current study will be useful for both implementation strategies and intervention theory building.

## Data Availability

Not applicable.

## Abbreviations

App: Smartphone application
VR: Virtual reality
RCT: Randomized controlled trial
ANCOVA: Analysis of covariance

## Footnotes

1 All custom software development, design and illustrations by Orb Amsterdam (www.orbamsterdam.com) and Studio Barbaar (https://studiobarbaar.com/).

2 Participants in the VR condition set a month goal at the start of the first VR session so that participants in all three conditions will have had an equal amount of time to work towards their month goal when completing the post measurement questionnaire.

3 The avatar does not unblur completely, as the future is open to changes.

4 In the second VR session participants describe a personal situation to their present self instead of reading questions from cards.
